# Towards a model of uncertainty distress in the context of Coronavirus (COVID-19)

**DOI:** 10.1017/S1754470X2000029X

**Published:** 2020-07-07

**Authors:** Mark Freeston, Ashley Tiplady, Lauren Mawn, Gioia Bottesi, Sarah Thwaites

**Affiliations:** 1 School of Psychology, Newcastle University, Newcastle upon Tyne, NE1 7RU, UK; 2 Newcastle Hospitals Occupational Health Service, Regent Point, Regent Farm Road, Gosforth NE3 3HD, UK; 3 Psychology in Healthcare, Royal Victoria Infirmary, Queen Victoria Road, Newcastle Upon Tyne NE1 4LP, UK; 4 Department of General Psychology, University of Padova, via Venezia 8, 35131 Padova, Italy

**Keywords:** Coronavirus, COVID-19, distress, uncertainty

## Abstract

The paper forms part of a series of papers outlining the theoretical framework for a new model of uncertainty distress (this paper), treatment implications arising from the model, and empirical tests of the model. We define uncertainty distress as *the subjective negative emotions experienced in response to the as yet unknown aspects of a given situation*. In the first paper we draw on a robust body of research on distinct areas including: threat models of anxiety, perceived illness uncertainty and intolerance of uncertainty. We explore how threat and uncertainty are separable in anxiety and how we can understand behaviours in response to uncertainty. Finally, we propose a clinically, theoretically and empirically informed model for uncertainty distress, and outline how this model can be tested. Caveats, clinical applications and practitioner key points are briefly included, although these are more fully outlined in the treatment implications article. While we outline this model in the context of novel coronavirus (COVID-19), the model has broader applications to both mental and physical health care settings.

**Key learning aims:**

(1) To define the concept of uncertainty distress.
(2) To understand the role of threat, over-estimation of threat, perceived uncertainty, actual uncertainty, and intolerance of uncertainty in distress maintenance.
(3) To understand how people may behave in response to uncertainty distress.
(4) To describe a model of uncertainty distress.

## Rationale

*The intriguing and worrisome characteristic of an emerging infectious disease is that the precise cause is at first unknown. This uncertainty in itself may increase the level of psychosocial morbidity*. Sim and Chua (2004)

This aim of this article is to attempt to link together three complementary but distinct literatures with the goal of developing a clinically, theoretically and empirically informed model for understanding and managing uncertainty distress. We are referring to uncertainty distress rather than to anxiety (health anxiety or other forms anxiety), stress, worry or burden because although the framework we propose has implications for all of these, the emphasis in this article is on dimensions of uncertainty rather than threat or the broader impact. Although this article is written in the context of Coronavirus, the ideas were based on work developing and testing treatments for the anxiety disorders (Mofrad and Tiplady, 2019; Tiplady *et al*., 2017) and further developed with reference to (amongst others) caregivers of dementia, caregivers facing acute and potentially life-threatening paediatric health conditions, caregivers of adults with developmental difficulties, multi-disciplinary teams working with a range of problems under conditions of actual uncertainty, asylum seekers, other people living under conditions of psychosocial instability, long-term health conditions, and so on.

This is the first of several linked articles, laying out the theoretical background, and proposing the model. The later articles will describe the novel treatment implications that flow from the model and empirical tests of the model.

In its simplest form we would agree with Kuang (2017) that uncertainty is a ‘psychological state of “not knowing” (p. 199)’. We further agree that uncertainty can be understood either objectively, that is, the actual state of knowledge (or not) about the accuracy of predictions of what will happen that is usually expressed in probabilistic terms, and subjectively, as cognitive and affective responses. For this article, we define uncertainty distress as ‘*the subjective negative emotions experienced in response to the as yet unknown aspects of a given situation*’. Furthermore, the knowledge, information and certainty that is currently not available is highly desired, even though it may not exist. As well as anxiety in varying degrees from concern to panic, these emotions may include frustration, anger and rage at the unfairness or helplessness of the situation and may be accompanied by other emotions. In the short term these include actual regret (for choices made), guilt (based on actions or inaction) through a felt sense of responsibility or shame if one has come up short of one’s or others’ expectations, and sadness and grief through lost opportunities or loss. These emotions may already be experienced in the present if certain things are happening or have already happened, but they may also be anticipated in the future, depending on how events unfold over time.

These emotions will be accompanied by a range of mostly negative cognitions and cognitive processes, often experienced as involuntary, as well as a range of behaviours that seek to mitigate the uncertainty (and associated distress) in the short, middle or long term. Importantly, these cognitions and behaviours (see section on behaviours below), together with the subjective distress may have a significant impact on people’s lives and day-to-day functioning, as well as the people around them. Although the combination of distress and impact on functioning at a certain level would normally define a disorder, we are not proposing a new disorder. We are describing a process that is both transdiagnostic in mental health problems but also trans-situational in a wide range of real-life contexts, whether acute (e.g. serious medical problems of unknown origin and sudden onset), chronic (e.g. the slower variation or unfolding of chronic or degenerative diseases) or the simply novel (e.g. Coronavirus).

It should be noted that the as yet unknown aspects of a given situation will often, but not invariably, be accompanied by elements of threat, whether real, perceived, or over-estimated. However, it is both theoretically and practically possible that uncertainty distress can be experienced by some people in situations where the likelihood of threat or a negative outcome is objectively very low or even absent. The as yet unknown aspects of the situation then include a range of neutral and positive outcomes. In these cases, it is more clearly the unknown-ness that is distressing rather than the possibility of threat or danger.

## The current pandemic

The COVID-19 pandemic represents an exemplar uncertainty situation within which we can illustrate the uncertainty distress model. The first studies on psychological distress in response to Coronavirus appeared in March 2020. For example, Wang *et al*. (2020) reported on an online survey (conducted from 31 January to 2 February 2020) in China (194 cities, *n* = 1210). Over half (53.8% of respondents) rated the psychological impact of the outbreak as moderate or severe; 16.5% reported moderate to severe depressive symptoms; 28.8% reported moderate to severe anxiety symptoms; and 8.1% reported moderate to severe stress levels. This early example indicates the level of distress reported in response to this threatening and uncertain situation.

However, we have been here before. In a study of the MERS outbreak in South Korea in 2015, Yang and Cho (2017) concluded that among university students, risk perception was higher in women than men, and was not related to age or knowledge about MERS. Risk perception was higher among those with greater trust in the media, local government and non-governmental organisations, but interestingly was lower among those with greater trust in medical systems, central government and healthcare policy, greater optimism about health policy, and greater willingness to sacrifice. In their study, risk perception was measured by rating seven statements on a 5-point scale (*completely disagree* to *completely agree*). These statements were about the possibility of contracting MERS with or without contact, one’s health being damaged, severity of MERS, avoiding hospitals because of MERS, damage to community and likelihood of return in Korea.

We would propose that ‘risk perception’ in this case is actually the perception of threat, consisting implicitly of both the degree of personally salient harm (including harm to society in a predominantly collectivist culture) and the likelihood of harm. At the time of the study, it could also be argued that these statements all reflected degrees of uncertainty. There were degrees of uncertainty about the likelihood of contracting MERS with or without contact, the severity of MERS and impact on a given individual was variable, and the future impact and potential return of MERS were as yet completely unknown. This example from MERS provides us with an illustration of the potential interest in a framework for understanding uncertainty distress in such situations from the standpoint of threat and uncertainty. In the rest of this article, we argue that to understand uncertainty distress there is a case for simultaneously considering the following:

(1) Actual threat;
(2) Perceived threat including over-estimation of threat;
(3) Actual uncertainty;
(4) Perceived uncertainty and the influences on this;
(5) Intolerance of uncertainty.

In order to do this we will draw on three main bodies of work, namely models of anxiety that have threat at their core (e.g. Salkovskis, 1991; Salkovskis, 1996), perceived uncertainty as understood initially in the context of illness (e.g. Mishel, 1981; Zhang, 2017), and intolerance of uncertainty (e.g. Carleton, 2016; Freeston *et al*., 1994) and we will consider how their integration has important implications for understanding and potentially mitigating uncertainty distress.

## Threat models of anxiety

Cognitive behaviour therapists in the United Kingdom are familiar with models of anxiety disorders based on perceptions threat. These are often referred to as the second wave of CBT and include the Clark (1986) model of panic, the Salkovskis (1985) model of obsessive compulsive disorder (OCD) and the Clark and Wells (1995) model of social anxiety. Extending earlier work by Carr (1974) and Beck (e.g. Beck *et al*., 1985), Salkovskis (1991, 1996) proposed an integrated account across disorders that anxiety can be understood as a response to the perceived likelihood of actual danger multiplied by the perceived awfulness or cost of that danger mitigated by the sum of coping and rescue factors. Milne *et al*. (2019) provide a review of the development of these ideas and summarise the limited research that has directly tested this model. They conclude that while the full equation has rarely been tested with the denominator terms included (rescue and coping), there is good evidence for the two separate components of likelihood and cost and limited evidence that the multiplicative component of the model predicts anxiety over and beyond the additive effects of likelihood and cost. However, the key point is that likelihood and cost are both important.

In anxiety, there is an over-estimation of threat relative to the actual or real-world state of threat and this occurs across a range of anxiety disorders and other disorders that have an anxiety component (see Abramowitz and Blakely, 2020). The time frame or the imminence of the threat is also an important component. Building on earlier work by a number of authors, Hamm (2020) differentiates between anxiety and fear in terms of imminence with associated psychophysiological, neural and behavioural correlates. Anxiety in distal threat becomes fear as the threat approaches and becomes imminent. There are a number of individual difference or dispositional variables that are believed to increase a person’s tendency to over-estimate threat. For example, the Looming Cognitive Style refers to an enhanced tendency to perceive future threat as both increasing in magnitude and accelerating in time towards the person as time moves forward (Riskind *et al*., 2000). A meta-analysis of 141 effect sizes found that this style is more strongly associated with non-specific anxiety (*k* = 46, *n* = 7914, *r* = .32, CI: .29 to .36, *p* < .001, *I*^2^ = .59), social anxiety (*k* = 10, *n* = 4513, *r* = .41, CI: .35 to .46, *p* < .001, *I*^2^ = .00) and worry (*k* = 25, *n* = 4528, *r* = .38, CI: .32 to .46) than with depression (*k* = 36, *n* = 7882, *r* = .27, CI: .23 to .30, *p* < .001, *I*^2^ = .47) (Yeo *et al*., 2020). Likewise, in the case of panic disorder (PD), Olatunji and Wolitzky-Taylor (2009) reported that anxiety sensitivity (AS), the tendency to mistake bodily sensations related to anxiety as a harmful experience (see Taylor, 1999), was higher in PD patients than non-clinical controls (*k* = 17, *n* = 14,920, *d* = 1.78, CI: 1.38 to 2.19, *p* < .001, *Q* (16) = 442.01, *p* < .001). Furthermore, they present evidence that suggests that AS is specific to panic compared with other anxiety disorders (except post-traumatic stress disorder) and mood disorders.

## Perceived uncertainty

Originally developed within nursing research and so perhaps less familiar to cognitive behaviour therapists, Mishel’s (1988) uncertainty in illness theory defines illness uncertainty as ‘the inability to determine the meaning of illness-related events’ (p. 225). This may be due to the unpredictability of the symptoms/course of illness or a lack of clarity about, for example, diagnosis, prognosis, treatment options, likely efficacy of treatment, roles within the care team, services available, responsibilities of patients and caregivers, their ability to engage in treatment, etc. Lack of clarity can arise either because any one of these (a) is currently unknown, (b) is not clearly communicated, (c) is not understood by the recipient, or (d) there is conflicting or ambiguous information from different sources. In relation to the previous model of anxiety, perceived uncertainty in the illness context can be either about threat (i.e. the likelihood and seriousness or cost of illness outcomes) or about rescue factors (i.e. treatment). Prognosis and treatment efficacy may also contribute to imminence in the perception of threat.

Mishel’s (1981) Uncertainty in Illness Scale has been used extensively in a wide range of settings, although the dimensionality of the scale varies according to the study. A meta-analysis of 32 studies on anxiety and information management within a variety of illness contexts (Kuang and Wilson, 2017) found that illness uncertainty is strongly and positively associated with anxiety (*k* = 34, *n* = 3541, *r* = 439, CI: .371 to .503, *Q* = 232.10, *p* < .05, *I*^2^ = 85.78) and information avoidance (*k* = 6, *n* = 532, *r* = 411, CI: .344 to .473, *Q* = 3.99, not significant, *I*^2^ < .001) Another meta-analysis of caregivers of youth with a range of chronic conditions (Szulczewski *et al*., 2017) reported that patient illness uncertainty was associated with distress in both patients (e.g. anxiety: *k* = 5, *r* = .369, *p* = .006, 95% CI: .113 to .58, *I*^2^ = 84.13; psychological distress: *k* = 5, *r* = .39, *p* = .003, 95% CI: .143 to .603, *I*^2^ = 86.48; self-rated illness distress: *k* = 13, *r* = .242, *p* = .000, 95% CI: .113 to .364, *I*^2^ = 79.45) as well as caregivers (e.g. psychological distress: *k* = 5, *r* = .078, *p* = .589, 95% CI: –.203 to .347, *I*^2^ = 65.42; illness related distress: *k* = 4, *r* = .161, *p* = .013, 95% CI: .034 to .283, *I*^2^ = 18.09). The converse was also true for some outcomes whereby caregiver uncertainty was associated with caregiver distress (e.g. anxiety: *k* = 9, *r* = .427, *p* = .000, 95% CI: .119 to .611, *I*^2^ = 92.93; and psychological distress: *k* = 8, *r* = .311, *p* = .018, 95% CI: .055 to .529, *I*^2^ = 90.47). Thus, there are robust findings linking perceived uncertainty as operationalised by the Uncertainty in Illness Scale to both patient and caregiver anxiety and psychological distress. Kuang (2017) further argues that perceived uncertainty goes beyond the context of illness and provides evidence for different contexts such as close and romantic relationships and organisational uncertainty.

## Intolerance of uncertainty (IU)

Since the publication of the Intolerance of Uncertainty Scale (IUS) in the early 1990s by a group at Université Laval in Québec (Freeston *et al*., 1994), IU has essentially been implicitly defined by what the scale measures (see Birrell *et al*., 2011). At that time the Laval team were attempting to identify the key cognitive feature of generalised anxiety disorder (GAD), equivalent in status to catastrophic misinterpretation of body sensations in panic (Clark, 1986) and inflated responsibility in OCD (Salkovskis, 1985). Playing a clinical hunch, they developed the IUS which has since proved to be helpful in understanding and treating GAD. The IUS is also increasingly utilised as a measure and predictor of clinical symptoms transdiagnostically across disorders (e.g. McEvoy *et al*., 2019), behavioural responses such as a specific role in maintaining threat bias during extinction (e.g. Morriss *et al*., 2019b), and of alterations in task-based activation of several brain regions, including the amygdala, prefrontal cortex, dorsal anterior cingulate and anterior insula (e.g. DeSerisy *et al*., 2020).

Although there has been much debate about the dimensionality of the scale, there has been a general consensus that there are two factors (e.g. Birrell *et al*., 2011; Carleton *et al*., 2007; Carleton *et al*., 2012; McEvoy and Mahoney, 2011; Sexton and Dugas, 2009). The first dimension, variously called *desire for predictability* or *prospective IU* consists mostly of items about wanting certainty. The second, variously called *uncertainty paralysis* or *inhibitory IU* consists of items about people getting stuck when faced with uncertainty. More recently, there is an emerging consensus from a series of recent psychometric studies using bi-factor analysis that the IUS-12, the shortened version of the original 27-item IUS (Carleton *et al*., 2007) is best understood as a univocal or unidimensional measure. In other words, the construct measured by the IUS-12 can be conceptualised as a general IU factor explaining most of the reliable variance across the items. Thus, both *prospective IU* and *inhibitory IU* may contribute to the IU construct, but the general IU factor is likely to have higher utility than the two dimensions separately (see Bottesi *et al*., 2019b, for review and additional evidence).

The definitions of IU, largely from the authors associated with the original Laval team, have varied over time (see Carleton, 2012, for a review). However, Carleton (2016) went beyond the reworking of the scale’s content and proposed a definition as ‘an individual’s dispositional incapacity to endure the aversive response triggered by the perceived absence of salient, key, or sufficient information, and sustained by the associated perception of uncertainty (p. 31)’. We would not disagree. However, we would state it slightly differently by first defining an uncertain event (or uncertainty) as a situation where the outcome is as yet unknown and where there is the possibility of a range of positive, neutral or negative outcomes. Then, intolerance of uncertainty is a tendency to be bothered or upset by the (as yet) unknown elements of a situation, *whether the possible outcome is negative or not*. Along with Kuang (2017), we emphasise the as yet ‘unknown-ness’ of the uncertain situation. Along with Carleton (2016), we emphasise the aversive response to the perception of uncertainty rather than beliefs about uncertainty or appraisals of the situation.

After Fridhandler (1986) we consider IU as a disposition: ‘A disposition is a property of some object (animate or inanimate) reducible to regular or expectable responses to certain circumstances or occurrences (Hempel, 1960). Formally, a disposition is expressible in one or a number of conditional sentences: “If x, then y”, where x is a set of conditions (circumstances or occurrences) and y is some behaviour’ (Fridhandler, 1986, p. 171). Thus IU is the (dispositional) tendency when encountering situations where the outcome as is yet unknown (but is potentially knowable in the fullness of time) to experience them as profoundly aversive (i.e. situational IU), regardless of the valence of potential outcome. In a specific uncertain situation, the aversiveness of this experience will cause people to engage in one or more uncertainty reducing behaviours which have the goal of reducing the uncertainty and the associated aversive internal state. While these behaviours may achieve their intended goal to a greater or lesser extent in the short term, they may also maintain or even increase intolerance of uncertainty both in that situation or other situations, including uncertain situations where there is no clear element of threat.

We would further propose that IU would increase the perceptions of greater uncertainty about the outcome and greater perceived severity of threat in these situations, although there will be additional determinants of these. Uncertainty reducing behaviours may also lead to perceptions of greater uncertainty and/or greater perceived severity of threat. For example, in relation to the 2009 H1N1 (swine flu) pandemic, a study of 1027 Canadian volunteers reported that people with high IU were more likely to perceive the pandemic as threatening and to report elevated levels of anxiety (Taha *et al*., 2014).

Therefore, we propose that IU is an individual difference variable that first leads people to experience uncertain situations as aversive, leads them to engage in uncertainty reducing behaviours, and then moderates their perceptions of uncertainty and threat. The more a person is intolerant to uncertainty, the more they will find uncertainty in that situation aversive in and of itself, the more they will perceive the situation as uncertain and threatening. In situations with real uncertainty and real threat, these three components together will lead to uncertainty distress often experienced (but not exclusively) as worry and anxiety.

## Understanding behaviour in response to uncertainty

In the build-up to the 2013 revision of the Diagnostic and Statistical Manual (DSM-5; American Psychiatric Association, 2013), as for most disorders, there had been work groups, field studies, expert consensus and articles considering possible changes for GAD under DSM-5. There was a great deal of discussion (see Andrews *et al*., 2010; Andrews and Hobbs, 2010; Starcevic *et al*., 2012; Starcevic and Portman, 2013) but eventually very little changed. However, there was a very interesting proposal, namely that there may be some GAD-specific behaviours. These were: ‘The anxiety and worry leads to changes in behavior shown by one (or more) of the following: (a) marked avoidance of potentially negative events or activities, (b) marked time and effort preparing for possible negative outcomes of events or activities, (c) marked procrastination in behavior or decision making due to worries, (d) repeatedly seeking reassurance due to worries (Andrews *et al*., 2010, pp. 141–142)’. Although not finally accepted, this is a first proposal that GAD, with worry at its core and with IU as an empirically based underlying driver of worry, has some specific behavioural signatures. Especially pertinent are (b) time and effort preparing for possible negative outcomes of events or activities and (c) procrastination in behaviour or decision making due to worries. Conversely, (a) avoidance of potentially negative events or activities and (d) repeatedly seeking reassurance, while both are a common feature of GAD, do not seem as specific to GAD compared with avoidance in general for anxiety disorders. Furthermore, reassurance seeking, although common across disorders (Kobori and Salkovskis, 2013), is often recognised as a particular feature of OCD. More specifically, during the H1N1 pandemic, Taha *et al*. (2014) reported that greater intolerance of uncertainty was related to lower appraisals of self- and other control, lower levels of problem-focused coping and higher levels of emotion-focused coping and more H1N1-related anxiety.

While cognitive behaviour therapists are familiar with the concept of safety seeking behaviours (see Rachman, 1984; Salkovskis, 1991; Thwaites and Freeston, 2005), the proposal that behaviours conceptualised as safety-seeking may rather be considered as uncertainty reducing may be less familiar (e.g. Askey-Jones *et al*., 2017). We proposed that there may be some patterns of behaviour that people use to manage uncertainty and/or the aversive experience of it (for a review, see Sankar *et al*., 2017).

Over-engagement consists of approach behaviours driven by attempts to attain certainty about outcomes in uncertain situations and so reduce aversive feelings of uncertainty. These include over-preparation, repeated questioning, prolonged internet searching, etc. Under-engagement represents avoidance-like behaviours motivated by attempts to disengage from future situations with uncertain outcomes and reduce aversive feelings associated with uncertainty. These behaviours include procrastination, distraction, information avoidance, etc. Impulsive behaviours seek to immediately eliminate uncertainty about outcomes in situations or especially the distress caused by uncertainty. This may include doing something without considering the consequences or prior planning simply to resolve it, i.e. to eliminate uncertainty, even when knowing that it may be a bad choice. ‘Dither’ behaviours can result in inaction due to hesitancy in choosing between at least two out of three courses of action, namely under-engagement (avoiding future uncertain situations), over-engagement (seeking future certainty), and impulsive (immediately reducing feelings of uncertainty), without really pursuing any of them. This can result in uncertainty paralysis. Finally, ‘Flip-Flop’ behaviour involves switching between at least two out of three courses of action, namely under- and over-engagement and impulsive behaviours over a longer time scale than dithering and can lead to a chaotic, disorganised and ineffective approach to a situation.

We developed a questionnaire, called Intolerance of Uncertainty Behaviours in Everyday Life (IUBEL; Clifford *et al*., 2015) based on the initial proposal of uncertainty reducing behaviours. Clifford (2014) reported that an individual’s repertoire of behaviours varied according to different life domains (finance, social, recreational, health and moral) among 346 undergraduate students. However, when looking at the most personally salient domain, individuals who were high in IU would use either a broader repertoire of strategies utilised at least ‘rarely’ and/or a narrower repertoire of strategies at a higher frequency of use. Overall, those with high IU used some combination of a broader repertoire of behaviours and a higher frequency of a few preferred IU-reducing behaviours.

Using an experimental paradigm, 69 undergraduates underwent a laboratory uncertainty induction and assessment of uncertainty reducing behaviours (using IUBEL) specific to the uncertainty situation (Bottesi *et al*., 2019a). Results indicated dispositional inhibitory IU/uncertainty paralysis positively predicted the use of under-engagement strategies and negatively predicted the use of over-engagement strategies. Furthermore, prospective IU/desire for predictability and worry positively predicted over-engagement behaviours. These developments, albeit in a very early stage, represent a first attempt to develop an empirical base for uncertainty reducing behaviours.

## Are threat and IU separable in anxiety?

Grupe and Nitschke (2013) proposed an ‘uncertainty and anticipation model of anxiety’ (UAMA), integrating psychological and neurobiological accounts, and which identifies five processes that would be familiar to CBT therapists, namely inflated estimates of threat cost and probability, increased threat attention and hypervigilance, deficient safety learning, behavioural and cognitive avoidance, and heightened reactivity to threat uncertainty. Two processes are critical to the current discussion: inflated estimates of threat cost and probability, and heightened reactivity to threat uncertainty. Although they propose that the anterior insula (AI) is implicated in both, importantly they distinguish between inflated estimates of threat cost and probability, associated with disruptions to the dorsomedial prefrontal cortex, rostral cingulate, orbitofrontal cortex and ventral striatum, whereas in heightened reactivity to threat uncertainty, AI dysfunction is associated with increased intolerance of uncertainty and contributes to bed nucleus of the stria terminalis (BNST) and amygdala hyperactivity and other midbrain and brainstem activity.

A more recent review extended some of these ideas and concluded that IU is associated with heightened reactivity to uncertainty as shown by greater activity of the anterior insula and amygdala, a mixed pattern of startle responses to uncertain threat and deﬁciencies in safety learning (Tanovic *et al*., 2018). Furthermore, Morriss *et al*. (2019a) identified three broad categories of uncertainty (basic threat and reward uncertainty, decision-making under uncertainty, and associative learning under uncertainty) and reviewed 87 studies using functional MRI (fMRI): basic threat and reward uncertainty. They examined the neural basis of each category and found shared and discrete patterns of neural activation for uncertainty, such as the insula and amygdala, depending on the category. Finally, consistent with Grupe and Nitzchke’s (2013) UAMA, in a meta-analysis of 23 studies (*n* = 466 with anxiety disorders and 508 healthy controls) the anxious group showed hypo-connectivity between the right amygdala and the dorsomedial pre-frontal cortex (Xu *et al*., 2019), one of the areas that the UAMA proposes is involved in inflated estimates of threat cost and probability. Furthermore, those with anxiety disorders also exhibited hypo-connectivity between the left amygdala and the ventromedial prefrontal cortex, one of the areas the UAMA proposes is involved in deficient safety learning. While we understand that there exists debate as to whether neural correlates contribute to a meaningful distinction at a mechanism level, the arguments outlined in combination with others are suggestive that there may exist a rationale to consider that threat and IU may be operating separately but also additively (or even interactively) in anxiety.

In more familiar CBT terms, Pepperdine *et al*. (2018) investigated appraisals of situational threat and IU in everyday uncertain situations, i.e. situations with an as yet unknown outcome that could range from the mildly negative to the mildly positive among a community sample (*n* = 224). Participants responded to more positive and more negative variants of the same situation. They reported that higher scores on the IUS were related to the perception of threat and uncertainty in both mildly positive and mildly negative situations, with the greatest contribution from being bothered by uncertainty. This was true in both the positive and negative situations. These results remained after controlling for the salience of the situations, trait optimism and trait pessimism. These findings support the notion that IU is not simply threat perception but rather an aversive response to uncertainty, although it also increases the perception of threat.

In a further study by Milne *et al*. (2017), 295 community adults (75% were 25 years or older), participated in a vignette-based study. Thirty per cent met criteria for elevated anxiety. They chose a personally salient concern, mapping to GAD-like concerns (44%), socially anxious concerns (31%), health anxious concerns (13%), and obsessive compulsive concerns (12%). Salience was rated by 70% as ‘very salient or greater’ (7 or above on a 1–10 to scale). After controlling for situation, salience and time period (imminence), both situational threat and IU contributed unique variance to situational anxiety across concerns, but IU contributed more. The exact strength of the relationship of situational threat and IU with anxiety varied according to the situation.

Together, these findings are supportive of the separate but additive contributions of threat and IU in both everyday and personally salient anxious situations, with cost/awfulness and likelihood the key elements of threat perception (cost/awfulness) and IU (perceived uncertainty and dislike of the uncertainty) the key elements of IU. Furthermore, cost is probably the most important part of threat, and dislike of uncertainty is probably the most important part of IU.

## A model of uncertainty distress

**Figure 1.**
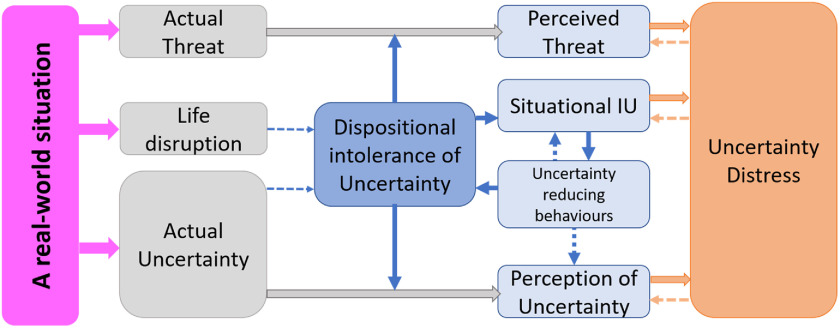
A model of uncertainty distress in response to a real-world situation.

Figure 1 presents a schematic representation of uncertainty distress. Actual threat and actual uncertainty are ‘real-world’ variables that reflect the state of what is known and not known about a given situation at a specific point in time. Intolerance of uncertainty is both a dispositional tendency to react to uncertain situations and as situational IU, the aversive experience in response to the specific situation. Life disruption refers to the effects of the real-world interference in a person’s life as distinct from a specific instance of uncertainty (e.g. changes to the normal everyday patterns and routines). It is proposed that over the short to medium term (and beyond a specific instance of uncertainty) this disruption will increase (dispositional) intolerance of uncertainty.

The key proposition is that dispositional intolerance of uncertainty will moderate both perception of threat and perception of uncertainty as well as lead to situational intolerance of uncertainty. In turn, situational intolerance of uncertainty will lead to uncertainty-reducing behaviours that will either maintain or increase both dispositional and situational intolerance of uncertainty. These behaviours may also contribute to the perception of uncertainty. Finally, perceived uncertainty, perceived threat and situational IU collectively contribute to the experience of uncertainty distress. Like many CBT models, it is assumed to be recursive, that is a ‘vicious circle’ model, where uncertainty distress will further increase perceived threat, perceived uncertainty and situational IU. In this representation, all three are of equal size, but it is easy to imagine that each could increase or decrease in importance, both as a function of time and situation.

## Testing the model

With the emergence of Coronavirus in early 2020, by late February it was obvious that it would soon become a pandemic and affect the UK, and was already affecting regions of Italy to a serious degree. So, rather than thinking hypothetically about threat and uncertainty as we had been doing over the previous year, we found ourselves, unfortunately, in the right place at the right time with a developing model to test in real time. We felt, having been trained and working actively as scientist practitioners, the obligation to do so. At the time of writing we, together with overseas collaborators, have been collecting data in English, Italian, Spanish and Greek (for project details, see Freeston *et al*., 2020). Although it will take us a while to collect and model the dynamic data (tracking date and locality) on uncertainty distress and psychological impact as the pandemic evolves, and conduct a follow-up study in several months, we will be analysing the first few days of the English language dataset and will submit an early test of the model for publication as soon as possible.

As developed in the preceding sections, the model states that uncertainty distress in a given situation results from actual or objective threat, perceived threat, actual uncertainty, perceived uncertainty, and situational intolerance of uncertainty, all specific to that situation. Furthermore, dispositional IU is a current predictor of situational IU and distress, and a moderator of perceived uncertainty and threat.

The key predictions are:

(1) Actual and perceived threat, actual and perceived uncertainty, and situation specific IU will all make unique contributions to variance in uncertainty distress and psychological impact.
(2) The unique contributions will remain (albeit weaker) when controlling for the general tendency to worry.
(3) Dispositional IU (IUS-12) will moderate the indirect/mediational path between actual uncertainty and perceived uncertainty and between actual threat and perceived threat (interaction terms).
(4) Uncertainty-reducing behaviour will mediate the relationship between situational IU and perceived uncertainty.

It will, of course be interesting to see whether there are any interactions between the main elements (actual and perceived threat, actual and perceived uncertainty, and situation specific IU) over and above the additive contributions of the key variables. For example, perceived uncertainty and perceived threat may interact, whereby the more uncertain a situation appears the greater the likelihood of the negative outcome must be adjusted, and probably in the direction of greater threat.

### Caveats

Although this model has drawn on three well-established existing literatures, each with an empirical basis, the proposed separate contributions may yet be proved to be essentially overlapping and not contribute either unique variance to uncertainty distress or lead to meaningful differences in how they should be addressed in practice. The theoretical developments about the origin of IU (e.g. Brosschot *et al*., 2016) and the increasing evidence that IU *vs* threat as well as IU *vs* trait anxiety may have separate neurobiological and behavioural correlates, would suggest that the line of reasoning proposed here that they may be separable may be worth pursuing. However, the distinctions proposed here need to be critically examined as the evidence develops and when stronger evidence becomes available.

It should be noted that this is a within-person model of how a dispositional variable (IU) leads to or moderates a series of processes leading to uncertainty distress. However, the majority of the evidence cited in this paper and the proposed tests of the model are from between-person studies and mostly cross-sectional studies. First there is need for better quality between-person (nomothetic) evidence in terms of longitudinal designs and experimental manipulations. Second, there is a long recognised inferential jump that evidence from between-person studies automatically applies to within-person models (see Molenaar, 2004) and within-person or idiographic studies are required (see Wright and Woods, 2020), despite the challenges of defining, operationalising and analysing such models (e.g. Fried, 2020).

## Clinical implications

The main implications for treatment will be laid out in the accompanying article where we will consider each of the components in turn and how each may be targeted in the context of real threat and uncertainty. Some will be familiar to CBT practitioners (e.g. those addressing perceived and over-estimation of threat), but require some adaptation. Others will be based on both evidence-based and theory-based arguments as to why a specific strategy, which may not be one that comes automatically to mind, may have some helpful application in specific circumstances (e.g. reducing perceived uncertainty and perceived threat through helping people reduce access to some types of information while potentially increasing access to others). Finally, some may at first glance seem counter-intuitive but may have a helpful role to play (e.g. helping people address the unsettling effects of life disruption and/or developing a greater tolerance of uncertainty).

Focusing on IU rather than on perceived uncertainty or perceived threat may represent a departure from the evidence base and we must consider the possibility that strategies that do so may not be effective or indeed may be unhelpful. Consequently, for a given individual, any better-established approaches should also be considered as to whether they may better fit the contributing factors and specific uncertainty distress experienced. To the extent that the current pandemic represents real uncertainty and real threat, it may be that the type of interventions from the perceived uncertainty literature may be more applicable than those from the perceived threat literature given that the former have been developed in situations with high objective uncertainty (and threat) rather than in the perceived threat literature where interventions have mostly, but not exclusively, been applied to anxiety disorders. The distinction is that in anxiety disorders, by definition the distress and behavioural responses are (although understandable) disproportionate or excessive as in over-prediction or over-estimation of threat.

The main implications from this proposal in the short term are for formulation. CBT practitioners use models to guide formulations as a way to make sense of and organise what can be an overwhelming amount of information. By starting to partition threat and uncertainty into relatively separate parts, we can start to make some sense of how the distress reaction can be understood as a series of interacting processes. Indeed, drawing out the model and filling it with factors contributing to each may help engage clients in a discussion that serves to educate and normalise the varying forms of distress experienced. It may also be beneficial to help clients distinguish what may be real and/or perceived and whether certain areas are more prominent than others, including mapping changes over time. First and foremost, however, this is a normalising model. Distress is expected given the degrees of threat and uncertainty that are present. That being said, by identifying which processes are potentially modifiable, practitioners can start to identify interventions that go beyond generic coping strategies and seek to mitigate distress in a targeted way.

## Key practice points

(1) Uncertainty distress is real and understandable, and current models of anxiety may only partially account for the level and breadth of distress experienced. Coronavirus (COVID-19) is an exemple of a real-world uncertain and threatening situation: it is a novel disease and an unprecedented challenge to individuals, health care and society in the broadest sense.
(2) For real-life events such as the pandemic, CBT models and treatment approaches need to both work within and expand beyond a traditional threat-based understanding of uncertainty distress.
(3) The uncertainty distress model is intended as a normalising model. Identifying different aspects of the model may help people understand the range of factors they are dealing with and how distress would be a normal reaction to their personal situations. It helps evidence a normal response to an abnormal experience rather than pathologising distress in response to major life experiences of uncertainty and threat.
(4) The proposed model includes perceived elements of a situation that are often factors considered in CBT in a mental health setting as well as incorporating the real or actual elements of a situation that are often encountered in physical health settings. Inclusion of both offers a unifying model that can be considered across mental and physical health settings.
(5) Each practitioner will have their own relationship to each part of the model in response to life experiences. To understand one’s own cognitions, behaviours and responses to distinct and overlapping factors in the model, it may be helpful to map and understand one’s own responses to both uncertainty and threat. This may be more ‘alive’ or visible in response to the current pandemic.
